# The mediating role of sleep, physical activity, and diet in the association between shift work and respiratory infections

**DOI:** 10.5271/sjweh.3896

**Published:** 2020-09-01

**Authors:** Bette Loef, Allard J van der Beek, Gerben Hulsegge, Debbie van Baarle, Karin I Proper

**Affiliations:** 1 Center for Nutrition, Prevention and Health Services, National Institute for Public Health and the Environment, Bilthoven, The Netherlands; 2 Department of Public and Occupational Health, Amsterdam UMC, Vrije Universiteit Amsterdam, Amsterdam Public Health Research Institute, Amsterdam, The Netherlands; 3 The Netherlands Organization for Applied Scientific Research, TNO, Leiden, The Netherlands; 4 Center for Immunology of Infectious Diseases and Vaccines, National Institute for Public Health and the Environment, Bilthoven, The Netherlands; 5 Department of Immunology, Laboratory for Translational Immunology, University Medical Center Utrecht, Utrecht University, Utrecht, The Netherlands

**Keywords:** acute respiratory infection, influenza-like illness, lifestyle behavior, mediation, mediation analysis, occupational health, shift worker

## Abstract

**Objectives:**

Shift work may be associated with an increased incidence of respiratory infections. However, underlying mechanisms are unclear. Therefore, our aim was to examine the mediating role of sleep, physical activity, and diet in the association between shift work and respiratory infections.

**Methods:**

This prospective cohort study included 396 shift and non-shift workers employed in hospitals. At baseline, sleep duration and physical activity were measured using actigraphy and sleep/activity diaries, sleep quality was reported, and frequency of meal and snack consumption was measured using food diaries. In the following six months, participants used a smartphone application to report their influenza-like illness/acute respiratory infection (ILI/ARI) symptoms daily. Mediation analysis of sleep, physical activity, and diet as potential mediators of the effect of shift work on ILI/ARI incidence rate was performed using structural equation modeling with negative binomial and logistic regression.

**Results:**

Shift workers had a 23% [incidence rate ratio (IRR) 1.23, 95% CI 1.01–1.49] higher incidence rate of ILI/ARI than non-shift workers. After adding the potential mediators to the model, this reduced to 15% (IRR 1.15, 95% CI 0.94–1.40). The largest mediating (ie, indirect) effect was found for poor sleep quality, with shift workers having 29% more ILI/ARI episodes via the pathway of poorer sleep quality (IRR 1.29, 95% CI 1.02–1.95).

**Conclusions:**

Compared to non-shift workers, shift workers had a higher incidence rate of ILI/ARI that was partly mediated by poorer sleep quality. Therefore, it may be relevant for future research to focus on perceived sleep quality as an underlying mechanism in the relation between shift work and increased infection susceptibility.

Workers employed in healthcare and various other occupational sectors regularly work in shifts around the clock (1). Together, this group of shift workers makes up a considerable part of the workforce, with 21% of European workers, and as much as 41% of healthcare workers, working in shifts (1). Nonetheless, shift work can be considered an occupational hazard because it has been linked to an increased risk of multiple diseases (2), such as cardiovascular diseases (3), metabolic syndrome (4), and diabetes mellitus type 2 (5). In addition, we recently found in a prospective cohort study that shift workers in healthcare had 20% more respiratory infections than non-shift workers, indicating that shift workers may also be more susceptible to infections (6). As respiratory infections, such as common cold and influenza-like illness, are responsible for a high burden of disease and substantial productivity loss (7), insight into possibilities to reduce this increased susceptibility in shift workers is needed.

Several possible explanations for the health effects of shift work have been proposed, including the behavioral pathways sleep, physical activity, and diet (8–10). Previous studies have shown shift workers to experience more sleep disturbances and have poorer dietary and physical activity habits (11–13). Engaging in these unhealthy behaviors has been found to be associated with increased infection susceptibility (14–16), and may, therefore, mediate the association between shift work and respiratory infections. However, research is currently lacking on the mediating role of behavioral pathways for health in general and infection susceptibility in particular. Studying this mediating role could contribute to the understanding of how shift work is linked to increased susceptibility to infection. Therefore, the aim of the current study was to examine the mediating role of sleep, physical activity, and diet in the association between shift work and respiratory infections.

## Methods

### Study design

The Klokwerk+ study is a prospective cohort study that aims to examine the effects of shift work on infection susceptibility and body weight gain, and the mechanisms underlying these health effects (17). In Klokwerk+, healthcare workers from different hospitals in The Netherlands used a smartphone application to report their influenza-like illness/acute respiratory infection (ILI/ARI) symptoms on a daily basis between September 2016 and June 2017. At the baseline measurement in September–December 2016, participants received the smartphone application, two accelerometers and a sleep/activity diary to measure sleep and physical activity for seven days, and a food diary to keep for three days. Furthermore, participants completed a questionnaire with questions about shift work status, lifestyle, and health. At the follow-up measurement in April–June 2017, another questionnaire was completed (17).

### Measures

*Shift work*. In the baseline and follow-up questionnaire, healthcare workers completed questions about their shift work status. Participants reported their current work schedule and whether they ever and currently worked night shifts (00.00–06.00 hours) and rotating shifts. Rotating shifts were defined as any work schedule rotating between day, evening, night and/or sleep shifts. Participants were considered shift workers if they worked rotating and/or night shifts (both at baseline and follow-up), and non-shift workers if they did not work rotating and night shifts for at least six months before baseline. For participants who changed their shift work status during follow-up (N=7), only the data collected up to that point in time was included.

*Respiratory infections*. The incidence rate of ILI/ARI episodes was used as measure for infection susceptibility (6). The occurrence of ILI/ARI episodes was measured using a smartphone application in which participants reported daily the presence or absence of the following symptoms: cough, sore throat, shortness of breath, runny/blocked nose, fever, malaise, hoarseness, and coughed-up mucus. An ILI/ARI episode was defined as having ≥2 symptoms on the same day or ≥1 symptom on two consecutive days (6). An episode ended when no symptoms were reported for two consecutive days. Sneezing and wheezing were also included in the symptoms list, but these symptoms were excluded from the definition of ILI/ARI to prevent classifying allergy symptoms as ILI/ARI. The application was initially developed to detect parent-reported cases of ILI in children and it appeared to be a useful tool for prospective studies. For the Klokwerk+ study, it was further adjusted to make it applicable for the measurement of ILI/ARI among adults (6).

*Potential mediators: sleep, physical activity, and diet*. To determine sleep and physical activity levels, participants were instructed to wear triaxial accelerometers (Actigraph GT3X devices, Actigraph, Pensacola, FL, USA) that were taped to their thigh, for 24 hours/day, for seven consecutive days (18). During these days, participants kept a diary in which work, sleep, and non-wear times were reported. Data from the accelerometers was analyzed using Acti4 software (NRCWE, Copenhagen, Denmark, and BAuA, Berlin, Germany). This software uses validated algorithms to estimate the time spent in the following body postures and physical activity types: sedentary (sitting/lying), standing, walking, running, stairclimbing, and cycling (19). First, periods not covered in the diary and non-wear time were excluded from data analysis. Next, based on sleep onset and offset times reported in the diary and corresponding sedentary periods in the accelerometer data, sleep duration was calculated. For every participant, the number of days with short (<7 hours/days) and long (≥9 hours/day) sleep duration were assessed (20, 21). Because not all participants wore the accelerometers for the same amount of time, the percentage of days with short and long sleep duration was calculated. Sleep quality was measured using one question from the Pittsburgh Sleep Quality Index (PSQI) in which participants were asked to indicate how they would rate their overall sleep quality in the past month (very good, fairly good, fairly poor, very poor) (22). This measure was dichotomized as fairly/very poor versus fairly/very good sleep quality. To determine physical activity levels, time spent in the different physical activity types during waking hours was assessed with the accelerometer data and expressed as percentage of the total time (18). Percentages of walking, running, stairclimbing, and cycling were combined to form one measure for physical activity during leisure and one measure for physical activity at work.

To assess dietary behaviors, participants kept a food diary for three consecutive days (23). In the food diary, the type and amount of consumed foods and drinks were reported, as well as the time of day at which these were consumed. Within the Klokwerk+ study, the focus was on the frequency of eating episodes in order to compare meal patterns and meal balance between shift and non-shift workers. Therefore, the validated Food-Based Classification of Eating Episodes (FBCE) was used to classify the eating episodes of the participants (24). In short, based on the combination of products (eg, animal protein, starch, vegetables, fruits, fats, sugars) consumed at one moment in time, the number of consumed meals and snacks per day was determined (23).

### Covariates

The following factors were important covariates in the association between shift work and respiratory infections based on earlier analyses (6) and were therefore included in the mediation model as potential confounders: age, gender, occupation (nurse versus other healthcare worker), influenza vaccination status (ie, whether participants received the seasonal influenza vaccine), and general perceived health [measured on a 5-point Likert scale (excellent–bad)].

### Statistical analysis

Differences between shift and non-shift workers in ILI/ARI incidence rate, potential mediators, and covariates were calculated using independent t- and chi-square tests.

Because the continuous mediators did not show a linear relation with ILI/ARI incidence rate, they were dichotomized based on the median. For consistency, the same cutoffs were used for the same lifestyle behaviors. The cutoffs were >33% versus ≤33% of days having a short (<7 hours/day) and long (≥9 hours/day) sleep duration, >12% versus ≤12% of leisure/working time being physically active (ie, walking, running, stairclimbing, and cycling), and >3 versus ≤3 meals/snacks per day.

**Figure 1:**
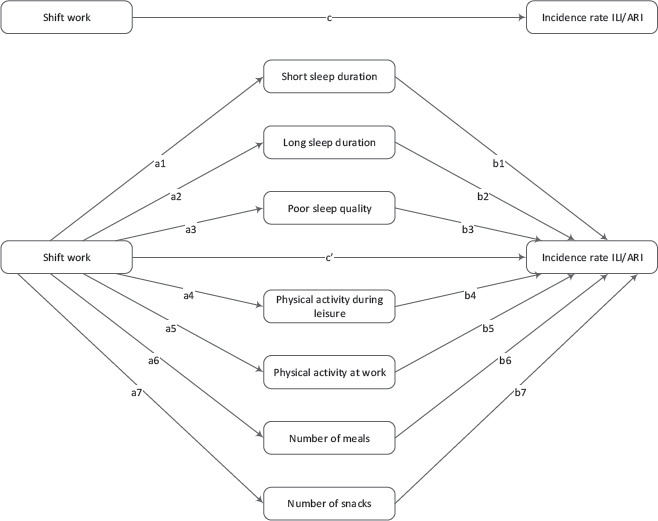
Multiple mediation model of the total effect (c) of shift work on influenza-like illness/acute respiratory infection (ILI/ARI) incidence rate, the indirect effects of sleep (a1-3, b1-3), physical activity (a4-5, b4-5), and diet (a6-7, b6-7), and the direct effect of shift work (c’) on ILI/ARI incidence rate.

The mediation analysis of sleep, physical activity, and diet as mediators of the effect of shift work on ILI/ARI incidence rate was conducted using structural equation modeling (SEM). SEM was chosen to construct the multiple mediation model because it is an efficient method to examine the pathways of different potential mediators simultaneously. The upper part of figure 1 shows the model of the total effect (c) of shift work on ILI/ARI incidence rate. The lower part shows the multiple mediation model of the indirect effects of sleep (a1-3, b1-3), physical activity (a4-5, b4-5), and diet (a6-7, b6-7), as well as the direct effect of shift work (c’) on ILI/ARI incidence rate that is independent of the included mediators and other covariates.

To calculate the estimates for the different paths, SEM was conducted with negative binomial regression for the c-path, b-paths, and c’-path to ILI/ARI incidence rate with the number of completed diaries as an offset variable (25), and with logistic regression for the a-paths to the dichotomous mediators. The indirect effect of each mediator was calculated as the product of the a-path and b-path (26). Next, a 95% bootstrap confidence interval (CI) using 5000 bootstrap resamples was calculated for each indirect effect to determine whether mediation was statistically significant (26, 27). The indirect effects and bootstrap CI were calculated using the untransformed regression coefficients of the logistic (a-paths) and negative binomial (b-paths) regression analyses. Subsequently, the results were back-transformed to create incidence rate ratios (IRR) by taking *e* (base of the natural logarithm) raised to the power of the regression coefficients.

In total, 396 participants (67%) had complete data on all potential mediators, ILI/ARI incidence rate, and covariates, and were therefore included in the current mediation analysis. Because there was a limited number of accelerometers available to measure sleep and physical activity, most cases of missing data were due to the fact that participants did not receive an accelerometer. To avoid possible bias due to exclusion of participants, missing values on sleep, physical activity, and diet were also imputed using multiple imputation with 33 imputation datasets (33% of participants had incomplete data) (28) in a sensitivity analysis.

SEM analyses were performed using Stata/SE, version 14.2 (StataCorp LLC, College Station, TX, USA) and multiple imputation was conducted using IBM SPSS Statistics, version 24.0 (IBM Corporation, Armonk, NY, USA).

## Results

### Study population

Of the 611 healthcare workers who enrolled in the Klokwerk+ study, 589 participants – who neither changed shift work status during follow-up nor stopped working in shifts in the 6 months before baseline – were included in the analysis of the association between shift work and ILI/ARI incidence rate in our earlier study (6). In the current study, 396 participants with complete data on sleep, physical activity, and diet were included for the multiple mediation analysis based on complete cases. Shift workers were younger [40.4 (standard deviation (SD) 12.1) years versus 47.0 (SD 10.4) years] and more often nurses (81.3% versus 33.3%) than non-shift workers (table 1). Shift workers reported on average more ILI/ARI episodes [3.5 (SD 2.4) episodes] than non-shift workers [2.9 (SD 1.8) episodes]. The largest differences between shift and non-shift workers in sleep, physical activity, and diet were found in short sleep duration and physical activity at work. Table 1 shows that 30.3% of shift workers frequently had a short sleep duration compared to 13.0% of non-shift workers. Furthermore, 53.5% of shift workers were highly physically active at work, compared to 24.6% of non-shift workers.

**Table 1:** Characteristics of the study population stratified for shift workers and non-shift workers. [ARI=acute respiratory infection; ILI=influenza-like illness; SD=standard deviation].

	Shift workers (N=327)		Non-shift workers (N=69)	
	
	%	Mean (SD)	%	Mean (SD)
Age (years)		40.4 ^a^ (12.1)		47.0 ^a^ (10.4)
Gender (female)	88.4		84.1	
Occupation (nurse)	81.3 ^a^		33.3 ^a^	
Influenza vaccination (yes)	14.1		21.7	
General perceived health (very good/excellent)	45.6		33.3	
Short sleep duration (>33% of days ^b^)	30.3 ^a^		13.0 ^a^	
Long sleep duration (>33% of days ^b^)	56.3 ^a^		42.0 ^a^	
Sleep quality (fairly/very poor)	18.3		10.1	
Physical activity during leisure (>12% of leisure time ^b^)	38.8		47.8	
Physical activity at work (>12% of working time ^b^)	53.5 ^a^		24.6 ^a^	
Number of meals (>3 per day ^b^)	13.5		18.8	
Number of snacks (>3 per day ^b^)	55.7		44.9	
Number of completed diaries		194.5 ^a^ (30.1)		200.0 ^a^ (15.7)
Number of ILI/ARI episodes		3.5 ^a^ (2.4)		2.9 ^a^ (1.8)

a Statistically significant difference (P<0.05) between shift and non-shift workers tested using independent-samples t-test and Chi-square test.

b Variables dichotomized based on the median values.

### Multiple mediation model

**Figure 2:**
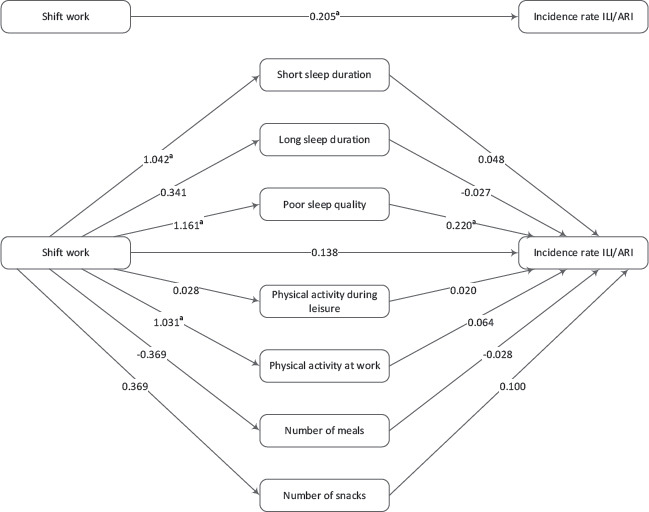
Multiple mediation model of the total effect of shift work on influenza-like illness/acute respiratory infection (ILI/ARI) incidence rate, the indirect effects of sleep, physical activity, and diet, and the direct effect of shift work on ILI/ARI incidence rate (complete case analysis, N=396). The values in the paths to the potential mediators represent untransformed coefficients from logistic regression analysis, and the values in the paths to incidence rate ILI/ARI represent untransformed coefficients from negative binomial regression analysis. Coefficients are adjusted for age, gender, occupation, influenza vaccination status, and general perceived health. ^a^ P<0.05.

Similar as reported previously for the total study population (6), shift workers had a higher incidence rate of ILI/ARI than non-shift workers in this subpopulation of Klokwerk+. The total effect of shift work on ILI/ARI incidence rate was 0.205 (95% CI 0.010–0.401), adjusted for covariates. This indicates that shift workers had a 23% [incidence rate ratio (IRR)=e^0.205^=1.23, 95% CI 1.01–1.49] higher incidence rate of ILI/ARI than non-shift workers [figure 2, table 2, supplementary material (www.sjweh.fi/show_abstract.php?abstract_id=3896) table S1]. After adding the potential mediators to the model, the direct effect of shift work on ILI/ARI incidence was 0.138 (95% CI -0.062–0.338) (IRR 1.15, 95% CI 0.94–1.40). Compared to non-shift workers, shift workers had a 2.84 times higher odds of frequently having a short sleep duration (95% CI 1.26–6.39) and a 3.19 times higher odds of having poor sleep quality (95% CI 1.27–8.01) (table 2). The odds ratio (OR) of having a high physical activity level at work was also higher in shift workers compared to non-shift workers (OR 2.80, 95% CI 1.47–5.34). Regarding the associations between the potential mediators and ILI/ARI incidence rate (b-paths), table 2 shows that self-reported poor sleep quality (IRR 1.25, 95% CI 1.05–1.47) was associated with a statistically significantly higher ILI/ARI incidence rate.

**Table 2:** Path coefficients [expressed as odds ratios (OR) and incidence rate ratios (IRR)] of sleep, physical activity, and diet on the association between shift work and ILI/ARI incidence rate (complete case analysis, N=396) ^a^ [ARI=acute respiratory illness; CI=confidence interval; ILI=influenza-like illness].

	a-paths (shift work -> mediator)		b-paths (mediator -> ILI/ARI)		c’-path (direct effect)		c-path (total effect)	
			
	OR	95% CI	IRR	95% CI	IRR	95% CI	IRR	95% CI
Direct and total effect					1.15	0.94–1.40	1.23 ^b^	1.01–1.49
Short sleep duration	2.84 ^b^	1.26–6.39	1.05	0.91–1.22				
Long sleep durations	1.41	0.77–2.56	0.97	0.85–1.11				
Poor sleep quality	3.19 ^b^	1.27–8.01	1.25 ^b^	1.05–1.47				
Physical activity during leisure	1.03	0.56–1.89	1.02	0.89–1.17				
Physical activity at work	2.80 ^b^	1.47–5.34	1.07	0.94–1.22				
Number of meals	0.69	0.32–1.51	0.97	0.81–1.17				
Number of snacks	1.45	0.80–2.61	1.10	0.97–1.26				

a Adjusted for age, gender, occupation, influenza vaccination status, and general perceived health.

b P<0.05.

The indirect effects of sleep, physical activity, and diet on the association between shift work and ILI/ARI incidence rate are presented in table 3. Shift workers had a 29% higher ILI/ARI incidence rate than non-shift workers via self-reported poorer sleep quality (IRR 1.29, 95% CI 1.02–1.95). The indirect effects of short (IRR 1.05, 95% CI 0.89–1.31) and long (IRR 0.99, 95% CI 0.91–1.06) sleep duration, physical activity during leisure (IRR 1.00, 95% CI 0.95–1.06) and at work (IRR 1.07, 95% CI 0.92–1.28), and number of meals (IRR 1.01, 95% CI 0.91–1.13) and snacks (IRR 1.04, 95% CI 0.97–1.16) were small and not statistically significant.

**Table 3:** Indirect effects of sleep, physical activity, and diet on the association between shift work and ILI/ARI incidence rate (complete case analysis, N=396) ^a^ [ARI=acute respiratory illness; CI=confidence interval; ILI=influenza-like illness; IRR=incidence rate ratio].

	Indirect effects ^b^	

	IRR	95% CI
Short sleep duration	1.05	0.89–1.31
Long sleep duration	0.99	0.91–1.06
Poor sleep quality	1.29 ^c^	1.02–1.95
Physical activity during leisure	1.00	0.95–1.06
Physical activity at work	1.07	0.92–1.28
Number of meals	1.01	0.91–1.13
Number of snacks	1.04	0.97–1.16

a Adjusted for age, gender, occupation, influenza vaccination status, and general perceived health.

b Indirect effects are calculated by taking e (base of the natural logarithm) raised to the power of the product of the a-paths and b-paths (eg, e(a1×b1)=e(1.042×0.048)=1.05).

c P<0.05.

Missing data on sleep, physical activity, and diet was imputed to conduct the multiple mediation model on the total study population (N=589). Supplementary figure S1 and table S2 show similar path coefficients and indirect effects for imputed data analysis as for complete case analysis.

## Discussion

The aim of the current study was to examine the mediating role of sleep, physical activity, and diet in the association between shift work and respiratory infections, defined as ILI/ARI incidence rate. Healthcare shift workers had a 23% higher incidence rate of ILI/ARI than non-shift workers. After including the potential mediating factors, this higher incidence rate of shift workers compared to non-shift workers reduced to 15%, and did not remain statistically significant. The association between shift work and ILI/ARI incidence rate was mediated by poorer sleep quality among shift workers.

Among this group of healthcare workers, shift work was associated with a higher incidence rate of ILI/ARI than non-shift workers. Correspondingly, evidence from a recent mechanistic study in mice suggests that shift workers who experience circadian disruption may be at increased risk for respiratory infections (29). Two epidemiological studies that examined the relation between shift work and infectious diseases also found an increased incidence of infectious diseases among shift workers (30, 31), but one study reported that infectious diseases were more common among non-shift workers (32). Nonetheless, these three previous studies had cross-sectional designs, and more prospective studies are needed to give further insight into the association between shift work and infectious diseases.

The largest indirect effect was found for self-reported poor sleep quality, with shift workers having 29% more ILI/ARI episodes via a poorer sleep quality. Shift workers more often reported a very or fairly poor sleep quality in the past month than non-shift workers, and poor sleep quality was statistically significantly associated with an increased ILI/ARI incidence rate. Similarly, although no mediation analysis was performed, in an earlier study among employees with different work schedules, shift workers had worse sleep quality and a higher prevalence of common infections compared to day workers (31). Together, these results indicate that sleep quality may be an important mechanistic factor of increased infection susceptibility among shift workers. Immunological mechanisms could potentially explain the connection between sleep and infection risk, as sleep is likely to modulate immune function (14, 33). A study among Japanese workers also concluded that sleep quality, and not just quantity, may be associated with alterations in white blood cell count (34). Based on the results of the current study, a focus on improving perceived sleep quality among shift workers may be recommended. Nonetheless, to increase the impact of a focus on sleep quality in prevention strategies, future research should confirm the findings of the current study and examine if sleep quality also mediates the association between shift work and other health outcomes.

In the current study, the perceived quality of sleep seemed to play a larger role in infection susceptibility of shift workers than objectively measured sleep duration. Both short and long sleep duration were not mediators in the association between shift work and respiratory infections. Prior reviews have concluded that shift work is associated with a decrease in sleep duration (35, 36). We also found that shift workers more often had a short sleep duration. Because a long sleep duration was also more frequently observed among shift workers in table 1, it becomes apparent that especially a regular sleep duration of 7–8 hours/day is often lacking in shift workers. With respect to the association between mediator and outcome, short as well as long sleep duration was not associated with ILI/ARI incidence rate. Nonetheless, earlier studies among healthy adults found an increased susceptibility to common cold and pneumonia among short sleepers (37–39). Regarding long sleep duration, a recent review concluded that there is currently not enough evidence to link long sleep duration to infection risk (33). An explanation for the fact that we did not observe a relation between short sleep duration and ILI/ARI may be that, in two of these earlier studies, the occurrence of infection was experimentally induced and sleep was monitored prior to administration of the virus (37, 39), while our approach was to measure shift workers’ general sleep duration and monitor subsequent naturally occurring infections. As general sleep duration may not be very representative for sleep duration prior to naturally occurring infections, we may have underestimated the effect of short sleep duration on ILI/ARI incidence. Therefore, it would be interesting to study also more acute effects of engaging in shift work, its immediate effects on sleep duration, and the possible subsequent increase in infection episodes.

Based on the results of the current study, we found no evidence that physical activity is an underlying mediator linking shift work and ILI/ARI incidence rate. As found earlier, physical activity levels of shift and non-shift workers during leisure were similar, but shift workers were more physically active at work (18). However, an association between physical activity and ILI/ARI incidence rate, and thus the association between mediator and outcome, was lacking. Correspondingly, a Cochrane systematic review did not find an association between moderate-intense physical activity and ARI (40). Diet (ie, number of meals and snacks) also did not mediate the association between shift work and ILI/ARI incidence rate. However, meal and snack frequency is only one aspect of dietary habits. As different micronutrients (eg, vitamins) are required for an efficient immune response (15), it may be relevant for future studies to take into account these dietary factors as potential mediators in the association between shift work and infectious diseases. Nonetheless, snacking behavior and meal consumption may still be important mediators in those health effects of shift work that more strongly depend on the energy balance, such as obesity and cardiovascular diseases (3).

### Strengths and limitations

In the current study, a daily diary application on a smartphone was used to measure ILI/ARI incidence during an entire winter season, resulting in 92% of all possibly completed diaries being completed (6). The reported incidence of ILI/ARI in this study was considerably higher than incidences derived from the traditional Dutch surveillance method, because an infection episode did not have to be confirmed by a clinician (6). Nonetheless, for the aim of comparing incidence rates between shift workers and non-shift workers, we believe our data are suitable.

Another strength is that multiple potential behavioral mediators were included, because the relation between shift work and health is likely to be multifactorial. Yet, sleep quality was the only relevant mediator in the association between shift work and ILI/ARI incidence rate, and, although not statistically significant, the ILI/ARI incidence rate of shift workers compared to non-shift workers was still 15% higher after including potential mediators. Therefore, studies with larger sample sizes that include more and different potential mediators (eg, psychosocial and physiological factors, such as stress, light exposure, and immunological factors) may be needed to better understand underlying mechanisms linking shift work and infection susceptibility. In addition, an important assumption of the statistical model used in this study is that there is no unmeasured confounding or interaction of the exposure–mediator, mediator–outcome, and exposure–outcome relations. Currently, new mediation methodology is developing in which these assumptions can be further explored, which can be used in future work (41). As this is the first study using mediation analysis to study the role of lifestyle behaviors in the association between shift work and respiratory infections, more research is needed to replicate our findings.

To conduct mediation analysis, a longitudinal design is preferred, in which the exposure is measured before the mediator, and the mediator is measured before the outcome (41). Although in the current study, exposure and mediators were both measured at baseline, shift work exposure referred to performing shift work for ≥6 months prior to baseline. Therefore, we feel confident that shift work exposure was already present before the measurements of the current lifestyle mediators at baseline. Furthermore, our aim was to measure shift work as an exposure that is long-term, and more chronic in nature, and we think it is unlikely that physical activity, sleep, and diet had a significant effect on this chronic measure of shift work exposure. A strength of the current study is its prospective design in which the outcome was measured after the exposure and mediators. Nonetheless, the measurement of the outcome started immediately after the baseline measurements of the mediators, without any time-lag. To address this issue, we conducted a sensitivity analysis in which we have only included episodes of the outcome measure ILI/ARI that occurred more than 30 days after the measurement of the mediators (supplementary figure S2 and table S3). This analysis provided the same results as the main analysis, which makes us more confident about the robustness of the findings.

In the current study, the objective measurements of the mediators were collected in a one-week time window. The use of objective instead of subjective measurements of sleep and physical activity is a strength of our study and provides us with valuable information to compare lifestyle behaviors between shift and non-shift workers on a group level. However, on the individual level, lifestyle behaviors may vary from one week to the next. For future research, it would therefore be useful to collect more repeated measurements of these lifestyle behaviors. Our aim was to study the effect of long-term, chronic shift work exposure, via general lifestyle behaviors, on ILI/ARI incidence. Nonetheless, collecting more repeated measurements of lifestyle as well as specific work schedule prior to the measurements of lifestyle, may give an opportunity to study acute effects of working night shifts on lifestyle and possibly subsequent immediate changes in respiratory infection risk. This would require a different design to take into account possible reversed causality, in which the occurrence of the infection episodes may influence lifestyle behaviors. To do so, a within-subject design comparing the effects of working night shifts and day shifts on lifestyle and infection risk within the same subjects may be suitable.

Approximately 20% of values on sleep, physical activity, and diet variables were missing. Missing data were primarily due to the fact that participants did not wear an accelerometer as there were not enough accelerometers available for all participants. There were no statistically significant differences between participants with missing data on one or more of the potential mediators (N=193) and participants without missing data (N=396) in age, gender, or any of the other covariates. Furthermore, as the results based on multiple imputed data did not differ from the complete case analysis, the impact of missing data on the results of this study is considered limited.

Lastly, the results of the current study apply to healthcare workers, and results may be different for other occupational groups.

### Concluding remarks

Compared to non-shift workers, shift workers had a higher incidence rate of ILI/ARI that was partly mediated by poorer sleep quality. Although shift work was also associated with short sleep duration and more physical activity at work, these factors were not mediators in the association with ILI/ARI incidence. For future research, it may be relevant to focus on perceived sleep quality as underlying mechanism in the relation between shift work and increased infection susceptibility.

## Supplementary material

Supplementary material

## Protection of research participants

Approval of the study was obtained from the institutional review board of the University Medical Center Utrecht, Utrecht, The Netherlands on March 15, 2016 (study protocol number 16-044/D, NL56022.041.16). Informed consent was obtained from all participants.
