# Supplementary material for: The mediating role of sleep, physical activity, and diet in the association between shift work and respiratory infections
Source: Scand J Work Environ Health. 2020 Apr 7;46(5):516–24. doi: 10.5271/sjweh.3896 (PMC7737798; doi:10.5271/sjweh.3896)
Supplement: Supplementary material [file SJWEH-46-516-S001.pdf]

The mediating role of sleep, physical activity, and diet in the association between shift work and respiratory infections [1]

by Bette Loef, MSc, Allard J van der Beek, PhD, Gerben Hulsegge, PhD, Debbie van Baarle, PhD, Karin I Proper, PhD [2]

1. *Supplementary tables and figures*

2. *Correspondence to: Karin I Proper, PhD, Center for Nutrition, Prevention and Health* Services, *National Institute for Public Health and the Environment; P.O. Box 1, 3720 BA Bilthoven, The Netherlands. [E-mail: karin.proper@rivm.nl]*

Table S1. Path coefficients (untransformed odds ratios and incidence rate ratios) of sleep, physical activity, and diet on the association between shift work and ILI/ARI incidence rate (complete case analysis, n=396)[a]

	a-paths (shift work -> mediator)			b-paths (mediator -> ILI/ARI)			c'-path (direct effect)			c-path (total effect)		
	B	95%-CI		B	95%-CI		B	95%-CI		B	95%-CI	
Direct and total effect							0.138	-0.062	0.338	0.205[b]	0.010	0.401
Short sleep duration	1.042[b]	0.231	1.854	0.048	-0.098	0.195						
Long sleep duration	0.341	-0.258	0.941	-0.027	-0.160	0.106						
Poor sleep quality	1.161[b]	0.242	2.081	0.220[b]	0.053	0.388						
Physical activity during leisure	0.028	-0.583	0.639	0.020	-0.115	0.154						
Physical activity at work	1.031[b]	0.387	1.675	0.064	-0.067	0.195						
Number of meals	-0.369	-1.149	0.410	-0.028	-0.213	0.158						
Number of snacks	0.369	-0.220	0.958	0.100	-0.030	0.229						

ARI, acute respiratory illness; B, regression coefficient; CI, confidence interval; ILI, influenza-like illness.
[a] Adjusted for age, gender, occupation, influenza vaccination status, and general perceived health.
[b] $p<0.05$.

Table S2. Indirect effects of sleep, physical activity, and diet on the association between shift work and ILI/ARI incidence rate (imputed data analysis, n=589)[a]

	Indirect effects[b]		
	IRR	95%-CI	
Short sleep duration	1.01	0.88	1.17
Long sleep duration	0.98	0.93	1.02
Poor sleep quality	1.33[c]	1.07	1.91
Physical activity during leisure	1.00	0.97	1.04
Physical activity at work	1.06	0.96	1.20
Number of meals	1.01	0.95	1.09
Number of snacks	1.03	0.99	1.11

ARI, acute respiratory illness; CI, confidence interval; ILI, influenza-like illness; IRR, incidence rate ratio.

[a] Adjusted for age, gender, occupation, influenza vaccination status, and general perceived health.

[b] Indirect effects are calculated by taking *e* (base of the natural logarithm) raised to the power of the product of the a-paths and b-paths (e.g. $e^{(a1*b1)}=e^{(1.011*0.013)}=1.00$).

[c] $p<0.05$.

Table S3. Indirect effects of sleep, physical activity, and diet on the association between shift work and ILI/ARI incidence rate with a 30-day time-lag between measurements of mediators and outcome (complete case analysis, n=396)[a]

	Indirect effects[b]		
	IRR	95%-CI	
Short sleep duration	1.05	0.86	1.34
Long sleep duration	1.00	0.92	1.07
Poor sleep quality	1.30[c]	1.01	2.04
Physical activity during leisure	1.00	0.93	1.08
Physical activity at work	1.10	0.94	1.36
Number of meals	1.01	0.89	1.15
Number of snacks	1.03	0.96	1.14

ARI, acute respiratory illness; CI, confidence interval; ILI, influenza-like illness; IRR, incidence rate ratio.

[a] Adjusted for age, gender, occupation, influenza vaccination status, and general perceived health.

[b] Indirect effects are calculated by taking *e* (base of the natural logarithm) raised to the power of the product of the a-paths and b-paths (e.g. $e^{(a1*b1)}=e^{(1.042*0.050)}=1.05$).

[c] $p<0.05$.

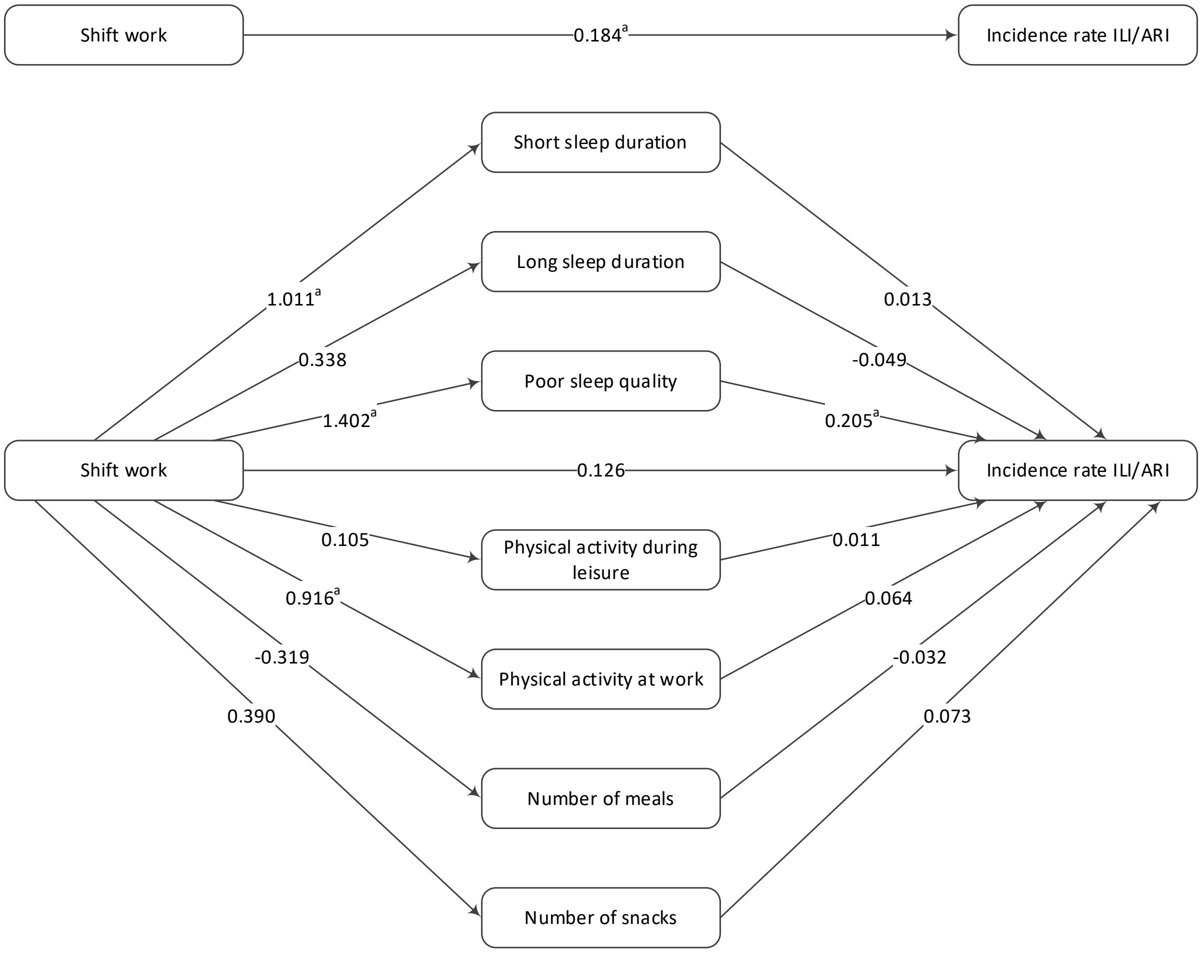

Figure S1. Multiple mediation model of the total effect of shift work on influenza-like illness/acute respiratory infection (ILI/ARI) incidence rate, the indirect effects of sleep, physical activity, and diet, and the direct effect of shift work on ILI/ARI incidence rate (imputed data analysis, n=589). The values in the paths to the potential mediators represent untransformed coefficients from logistic regression analysis, and the values in the paths to incidence rate ILI/ARI represent untransformed coefficients from negative binomial regression analysis. Coefficients are adjusted for age, gender, occupation, influenza vaccination status, and general perceived health. [a] $p<0.05$.

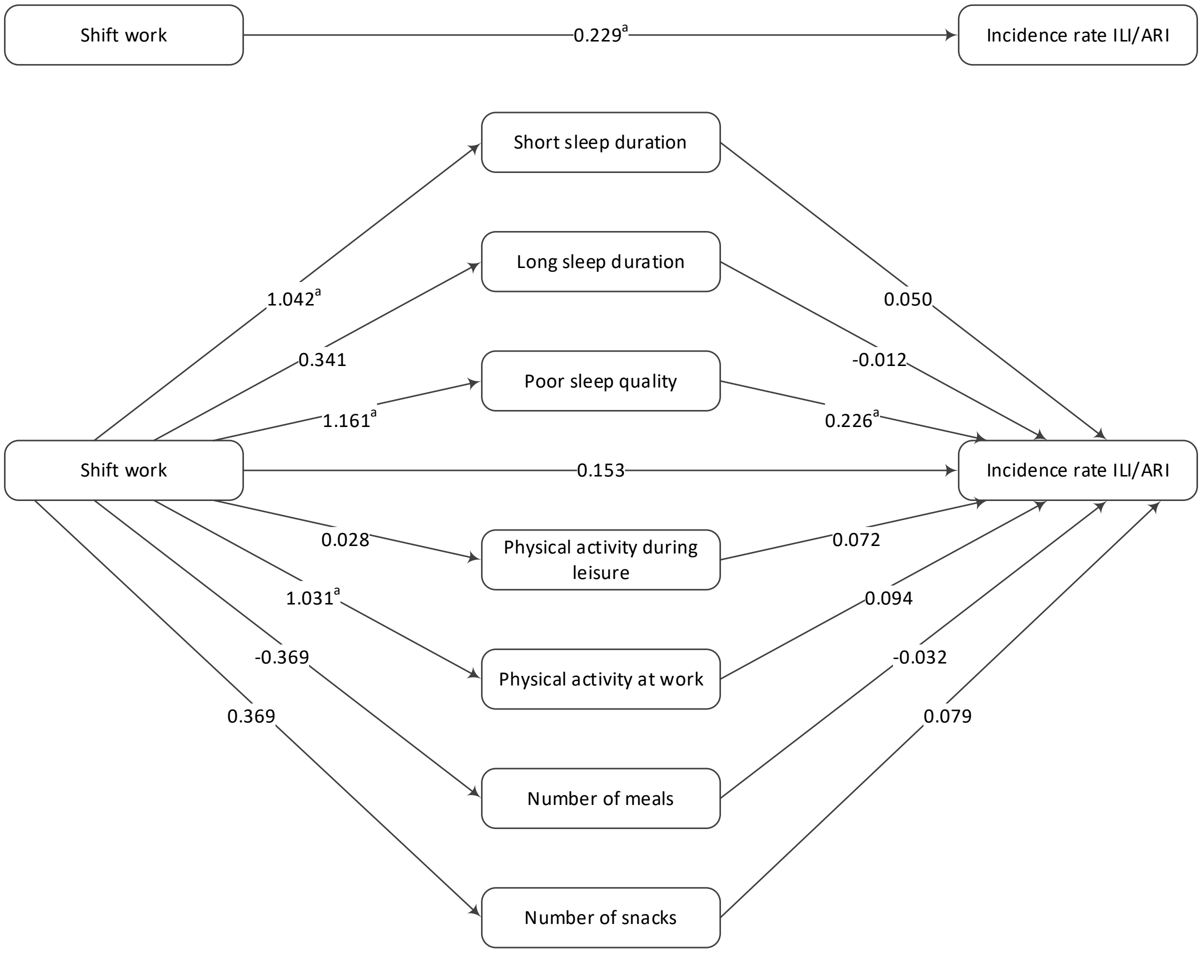

Figure S2. Multiple mediation model of the total effect of shift work on influenza-like illness/acute respiratory infection (ILI/ARI) incidence rate, the indirect effects of sleep, physical activity, and diet, and the direct effect of shift work on ILI/ARI incidence rate, with a 30-day time-lag between measurements of mediators and outcome (complete case analysis, n=396). The values in the paths to the potential mediators represent untransformed coefficients from logistic regression analysis, and the values in the paths to incidence rate ILI/ARI represent untransformed coefficients from negative binomial regression analysis. Coefficients are adjusted for age, gender, occupation, influenza vaccination status, and general perceived health. [a] $p<0.05$.
